# A maternal low protein diet has pronounced effects on mitochondrial gene expression in offspring liver and skeletal muscle; protective effect of taurine

**DOI:** 10.1186/1423-0127-17-S1-S38

**Published:** 2010-08-24

**Authors:** Ole Hartvig Mortensen, Hanne Lodberg Olsen, Lis Frandsen, Peter Eigil Nielsen, Finn Cilius Nielsen, Niels Grunnet, Bjørn Quistorff

**Affiliations:** 1 Department of Biomedical Sciences, Faculty of Health Sciences, University of Copenhagen, 2200 Copenhagen, Denmark; 2 Department of Cellular and Molecular Medicine, Faculty of Health Sciences, University of Copenhagen, 2200 Copenhagen, Denmark; 3 Department of Clinical Biochemistry, Rigshospitalet and Faculty of Health Sciences, University of Copenhagen, 2100 Copenhagen, Denmark

## Abstract

**Background:**

Low birth weight is associated with an increased risk of developing impaired glucose tolerance, and eventually type 2 diabetes in adult life. Gestational protein restriction in rodents gives rise to a low birth weight phenotype in the offspring.

**Results:**

We examined gene expression changes in liver and skeletal muscle of mice subjected to gestational protein restriction (LP) or not (NP), with or without taurine supplementation in the drinking water. LP offspring had a 40% lower birth weight than NP offspring, with taurine preventing half the decrease. Microarray gene expression analysis of newborn mice revealed significant changes in 2012 genes in liver and 967 genes in skeletal muscle of LP offspring. Taurine prevented 30% and 46% of these expression changes, respectively. Mitochondrial genes, especially those involved with oxidative phosphorylation, were more abundantly changed than other genes. The mitochondrial genes were mainly upregulated in liver, but downregulated in skeletal muscle, despite no change in citrate synthase activity in either tissue. Taurine preferentially rescued genes concerned with fatty acid metabolism in liver and with oxidative phosphorylation and TCA cycle in skeletal muscle. A mitochondrial signature was seen in the liver of NP offspring with taurine supplementation, as gene sets for mitochondrial ribosome as well as lipid metabolism were over represented in 4-week-old offspring subjected to gestational taurine supplementation. Likewise, 11 mitochondrial genes were significantly upregulated by gestational taurine supplementation in 4-week-old NP offspring.

**Conclusions:**

Gestational protein restriction resulted in lower birth weight associated with significant gene expression changes, which was different in liver and muscle of offspring. However, a major part of the birth weight decrease and the expression changes were prevented by maternal taurine supplementation, implying taurine is a key factor in determining expression patterns during development and in that respect also an important component in metabolic fetal programming.

## Background

Low birth weight is associated with increased risk of developing an abnormal metabolic phenotype such as obesity and type 2 diabetes [1] in later life [2] and is in humans associated with impaired insulin signaling in skeletal muscle [3-5], hepatic insulin resistance [6], and decreased insulin secretion [7] in adult life. However, the exact mechanism by which impaired fetal growth confers insulin resistance is unknown, although recently mitochondrial oxidative stress was proposed as a possible mechanism [8].

Animal models mimicking impaired fetal growth all display dysregulated glucose metabolism and altered insulin sensitivity in adult life [9]. Gestational protein restriction, where dams are fed a low protein (LP) diet during pregnancy, is one such model [9] and although most studies have focused on betal-cell dysfunction [9], an increase in peripheral insulin sensitivity in young animals [10] and a decrease in old animals [11,12] has been observed in offspring subjected to a maternal low protein diet.

Taurine, a sulfur-containing amino acid which does not enter protein synthesis, has a number of physiological functions such as conjugation with bile acids, osmotic pressure regulation in brain and antioxidant properties. Additionally, taurine functions as a chemical chaperone in conjugation with ursodeoxycholic acid, thereby relieving ER stress, and may be required for optimal mitochondrial protein synthesis, as taurine conjugation of one mitochondrial tRNA is required for optimal function tRNA [13]. Taurine has also been suggested to be involved in skeletal muscle fatigue, most likely due to a mitochondrial effect [14]  (for reviews, see [15,16]). Furthermore, taurine lowers blood glucose in type 2 diabetic patients [16,17] and has in rodents been shown to be able to prevent or delay development of insulin resistance induced by fructose-overfeeding in rodents [18]. Taurine ameliorates some of the harmful effects that gestational protein restriction confers upon the pancreas of the offspring by normalizing proliferation [19], vascularization, and decreasing sensitivity towards cytokines in pancreatic islets [20]. Collectively, these studies suggest that taurine has a profound impact on gene expression, a ‘reprogramming’ or rescuing effect, during fetal development, perhaps via epigenetic and/or organogenesis related mechanisms.

In the present study, we examined the effect of maternal taurine supplementation upon the offspring of gestational protein restriction by gene expression profiling of liver and skeletal muscle.

## Experimental procedures

### Animals

Virgin female (7-8 weeks old, N=3 per diet group) C57BL/6 mice (Taconic, Ejby, Denmark) were mated with C57BL/6 male mice. Following observation of a vaginal plug (gestation day 0), the mice were randomized into four different diet groups: Normal protein (20% casein; NP; Hope Farms 4400.00, Woerden, NL) or low protein (8% casein; LP; Hope Farms 4400.01), both with no detectable taurine, with or without 1% (w/v) taurine in the drinking water (tau, i.e. NP+tau or LP+tau) (synthetic taurine; Sigma-Aldrich, St. Louis, MO, USA).

The four different diet groups were considered largely isocaloric since taurine supplementation only marginally contributes to the calorie intake as taurine is not metabolized and cannot make up for the decreased intake of essential amino acids caused by the LP diet. Mice were kept in a 12-hour light/dark cycle. At day 19 the mice gave birth and newborn pups were weighed, killed by decapitation and liver and hind leg skeletal muscle quickly and quantitatively removed, weighed, quick frozen in liquid nitrogen and stored at -80 °C for further analysis. For the newborn mice, a total of 25 pups from 12 different dams were analyzed, with 5≤n≤7 pups per group, all groups being a mixture of male and female pups. For examination of an effect of taurine upon NP offspring, 3 animals from NP and NP+tau were sacrificed at 4 weeks of age, followed by decapitation, quick removal of liver and quadriceps muscle. Tissue was quick frozen in liquid nitrogen and stored at -80 °C until further analysis. All experimental procedures were approved by The National Committee on Animal Experimentation, Denmark and by the local animal facility at the University of Copenhagen, Denmark.

### RNA preparation

RNA was extracted from muscle and liver tissue using Trizol (Invitrogen, Carlsbad, CA, USA) as described by the manufacturer. In brief, tissue was homogenized in Trizol with a 5 mm steel bead, using a Qiagen Tissuelyzer (Qiagen). Upon lysis, RNA was extracted with chloroform and precipitated using isopropanol. Finally, RNA was subjected to cleanup using RNeasy columns (Qiagen). RNA quality was assured using a Bioanalyzer (Agilent, Santa Clara, CA, USA).

### Microarray experiment

Three randomly selected samples of RNA from each of the four dietary groups as well as from the 4-week-old NP and NP+tau groups were analyzed at the *RH Microarray Center* (Rigshospitalet, Copenhagen, Denmark). RNA labelling, Affymetrix Mouse 430 2.0 array (Affymetrix Inc., Santa Clara, CA, USA) hybridization, and scanning were performed according to the manufacturer’s instructions. Each pup used was from a different dam, with liver and muscle being analyzed from the same animal. It was ensured that all groups constituted either one male and two females or one female and two male pups. Arrays from liver and muscle were processed and analyzed separately as described below. Samples from the 4-week-old animals were also analyzed separately.

Raw CEL files from all groups were normalized using quantile normalization, and log2 probe expression values were obtained using RMA in one step using justRMA (R 2.7.0 and Bioconductor 2.2 [21]). Control probes and probes not present on at least one microarray (according to MAS5 absent present calls) were removed before further analysis. For statistical analysis of the remaining probes a strategy of pre-selecting probes for subsequent 2-way analysis of variance (ANOVA) was adopted. Using significance analysis of microarrays (SAM) [22], we selected genes that were different between the following pairs of groups with a false discovery rate (FDR) of less than 10%: NP vs NP+tau, NP vs. LP, NP+tau vs. LP+tau, and LP vs. LP+tau. The resulting list of significantly changed probes were then analyzed using a 2-way ANOVA model: log2(probe expression level) = protein taurine protein*taurine, with p<0.05 considered significant. Due to the previous SAM pre-selection, no adjustment for multiple testing was done. Finally, this list of probes was subjected to a student’s t-test examining whether or not there was a significant (p<0.05) difference between NP and LP. In case of multiple probes for the same gene, the probe with the best rescue effect (see below) was chosen, and in the case of multiple probes having the same rescue effect the probe with the highest average expression level in NP was chosen. The resulting list was annotated using Bioconductor 2.2 and manually curated, and genes considered unknown (expressed sequence tags, putative genes, RIKEN cDNAs etc.) were removed.

Changes in gene expression levels caused by the maternal LP diet were considered fully rescued by taurine when the following criteria assessed by students’ t-tests were met: 1) The gene expression level of LP and LP+tau must be significantly different (p<0.05), and 2) the gene expression level between NP and LP+tau must not be significantly different (p>0.05). Likewise, a gene was considered partially rescued if: 1) The gene expression level between LP and LP+tau was significantly different (p<0.05) and 2) LP+tau confer a change in the opposite direction of LP compared to NP.

No differences in gene expression were found when directly comparing NP or NP+tau diet offspring with LP+tau offspring using SAM (FDR<10%, data not shown) in both liver and skeletal muscle. Others have used this as proof of a total phenotype reversal [23], however the above more fine-grained analysis show only a partial rescue effect of taurine.

Cluster analyses were carried out on log2 expression values using dChip [24], clustering both samples and genes, standardized to row means, with precalculated correlation distances using average linkage. Over-representation of specific gene sets among the genes significantly changed by an LP diet, were estimated using DAVID 2008 [25] with probe ids for the unique genes as input compared against a list of probe ids for known unique genes without control genes and probes without a valid EntrezGene id as background. Fishers exact test was used to examine if taurine selectively rescued specific gene sets rather than having a general rescue effect on all genes. Gene annotations were carried out using information from DAVID 2008 and Genecards (http://www.genecards.org) [26]. Mitochondrial genes were assessed as genes with a GO cellular compartment term containing either mitochondrial or mitochondrion. All statistical analyses on microarray data were performed using R 2.7.0.

In order to detect changes in gene expression caused by gestational taurine supplementation in 4-week-old offspring (NP+tau vs. NP), 4-week samples were normalized by tissue as above and probes not present on at least one microarray (according to MAS5 absent present calls) were removed. Following this, a gene set enrichment analysis (GSEA) [27] was performed with the GO cellular compartments (CC) and KEGG genesets using standard parameters. Furthermore, a subset probeset consisting of mitochondrial genes (GO CC containing mitochon*) were subjected to a SAM analysis examining differences between NP and NP+tau in both 0- and 4- week animals, with an FDR of less than 10% considered significant. The data discussed in this publication have been deposited in NCBIs Gene Expression Omnibus(GEO, http://www.ncbi.nlm.nih.gov/geo) [28] and are accessible through GEO series accession number GSE12730 (newborn samples) and GSE20577 (4 week samples).

### Biochemical measurements

A 5% (wt/vol) homogenate of skeletal muscle tissue was prepared in a 2-ml Potter-Elvehjem glass-teflon homogenizer for 1 min at 0  °C in a buffer containing 25 mM glycyl-glycin, 150 mM KCl, 5 mM MgSO4, 5 mM EDTA, 1mM DTT, 0.02% BSA, and 0.1% Triton X-100, pH 7.5. The liver homogenate was prepared in the same buffer applying a Qiagen Tissuelyzer (Qiagen, Valencia, CA, USA) at 0 °C employing ice-cold racks, homogenizing for 3 times 1 min at 30 Hz. The homogenate was frozen in liquid nitrogen, thawed on ice, whirl mixed, and centrifuged at 22,000 g for 2 min at 4 °C. The supernatant was stored at -80 °C for determination of enzyme activities and protein concentration. Protein content was measured using BSA (fraction V) as standard [29]. Citrate synthase (CS, EC 4.1.3.7) activity was determined as described previously [30] and expressed relative to the total protein content as units per mg protein.

### Quantitative real-time RT-PCR

Reverse transcription (RT) reactions were performed using random hexamers on 2 µg RNA using an RT kit (Applied Biosystems, Foster City, CA) in a reaction volume of 100 µl. The resulting cDNA product was stored at -20 °C until further analysis. The primers and probes for all genes and the 18S rRNA endogenous control were pre-developed TaqMan probes and primer sets from Applied Biosystems: ACSS2 (acyl-CoA synthetase short-chain family member 2; Mm00480101_m1), COX7A1 (cytochrome c oxidase, subunit VIIa 1; Mm00438296_m1), COX7C (cytochrome c oxidase, subunit VIIc; Mm01340476_m1), CS (citrate synthase; Mm00466043_m1), EGFR (epidermal growth factor receptor; Mm00433023_m1), FST (Follistatin; Mm00514982_m1), GLDC (glycine decarboxylase; Mm00506891_m1), GPD2 (glycerol phosphate dehydrogenase 2, mitochondrial; Mm00439082_m1), MH1 (myosin, heavy polypeptide 1, skeletal muscle, adult; Mm01332489_m1), MYBPC2 (myosin binding protein C, fast-type; Mm00525419_m1), PDK4 (pyruvate dehydrogenase kinase, isoenzyme 4; Mm00443325_m1), SPP1 (secreted phosphoprotein 1; Mm00436767_m1), UCP3 (uncoupling protein 3 (mitochondrial, proton carrier); Mm00494074_m1) PGC-1α (peroxisome proliferator receptor γ coactivator 1; Mm00447183_m1). All assay reagents were from Applied Biosystems. The mRNA levels of all genes and the endogenous control, 18S rRNA, were determined by real time RT-PCR using an ABI PRISM 7900 sequence detector (Applied Biosystems). The amplification mixtures were amplified according to standard conditions using 50 cycles in a 10 µl volume in triplicate. The relative mRNA content of both the target and the endogenous control gene was calculated from the cycle threshold values by using a standard curve constructed from a serial dilution of aliquots of cDNA pooled from all the samples. The relative expression levels of all genes were determined by normalization to the endogenous control, 18S rRNA, which was found not to differ between diet groups.

### Statistics

Birthweight, liver and muscle weight as well as all enzyme activity data exhibited a normal distribution, and data are presented as means ± standard error of the mean (SEM). All microarray and mRNA data were log-transformed in order to obtain a normal distribution, and data are presented as geometric means with 95% confidence intervals. Statistical analyses were carried out using a 2-way ANOVA with Bonferroni corrected students’ t-tests as post hoc tests. All statistical analyses were performed using SAS 9.1.2 (The SAS Institute), except for the microarray analysis which was performed as described above. A p value < 0.05 was considered significant.

## Results

### Birthweight and citrate synthase enzyme activity in liver and muscle of newborn mice

Mouse dams were subjected to four different isocaloric diet regimes from day 1 of pregnancy until giving birth as described in materials and methods. The LP diet caused a ~40 % decrease (p<0.001) of the birthweight of the pups (Table 1). Supplementing the maternal LP diet with taurine reduced the birthweight loss by half (p<0.01) (Table 1). Both liver and skeletal muscle mass of newborn pups were decreased in proportion to body weight by the maternal LP diet (data not shown).

**Table 1:** Maternal low protein diet effects on body mass and citrate synthase activity in newborn mice.

Taurine	-	+	-	+	2-way ANOVA		
					
Protein	NP	NP	LP	LP	chow	taurine	c*t
Body mass (g)	1.23±0.03	1.15±0.05	0.72±0.05	1.01±0.06^**,*^	0.0001	0.037	0.0008
CS liver (mU/mg)	80.2±5.7	85.1±2.5	74.7±4.3	75.6±3.8	(0.0825)	ns	ns
CS muscle (mU/mg)	127.1±12.2	133.1±8.5	140.7±15.3	149.9±13.4	ns	ns	ns

Newborn mice subjected to different diet regimes *in utero* as described in materials and methods were weighed at birth and killed. Liver and skeletal muscle tissue were extracted, and citrate synthase (CS; mU/mg protein) enzyme activity measured as described. BM) Body mass, NP) normal protein (20% casein) or LP) low protein (8% casein) during pregnancy. Taurine) 1% taurine supplementation in the drinking water during pregnancy. Bonferroni corrected post hoc t-tests performed if chow*taurine (c*t) interaction was significant: ¶) p<0.001 NP vs. LP, **) p<0.01 LP vs. LP+taurine, *) p<0.05 NP vs. LP+taurine. All values are shown as means ± SEM. 5 ≤ N ≤ 7 per diet group.

As a measure of the mitochondrial fraction of tissue mass, citrate synthase (CS) specific enzyme activity and mRNA level [31] was determined in liver and skeletal muscle of the newborn pups. There was no difference between diet groups in CS enzyme specific activities (Table 1) or mRNA levels (see Additional file 1).

### Taurine partially rescues the effects of gestational protein restriction on gene expression levels in liver and skeletal muscle of newborn mice

Gene expression profiles of three randomly chosen newborn offspring samples from each diet group of both liver and skeletal muscle were analyzed using Affymetrix gene expression microarrays as described in materials and methods (Table 2).

**Table 2:** General data from the microarray analyses.

Description	Liver	Muscle
Total number of probes on the Affymetrix Mouse 430 2.0 gene expression array	45101	45101
Number of probes present on at least one array	28653	30966
Above without control probes or probes without a valid EntrezGene identifier	27300	29443
Probes significantly changed in SAM preprocessing (FDR<10%)	6047	1559
Probes significantly changed (p<0.05) in two-way ANOVA	3678	1284
Probes significantly different (p<0.05) between NP and LP	2827	1272
Unique genes significantly different (p<0.05) between NP and LP	2363	1110
Known genes significantly different (p<0.05) between NP and LP	2012	967
Known genes different between NP and LP and fully rescued by taurine	510	423
Known genes different between NP and LP and partially rescued by taurine	90	21
Total number of known genes rescued by taurine	600	444
Percentage known genes different between NP and LP rescued by taurine	30%	46%

A list of probe or gene numbers detailing the microarray gene expression analyses of liver and skeletal muscle of newborn mice, as described. NP) normal protein (20% casein) or LP) low protein (8% casein) during pregnancy. Table reprinted with permission from Wolters Kluwer Health / Lippincott Williams & Wilkins. [52]

In liver, the expression of a total of 2012 non-redundant transcripts, all encoding known genes, were significantly changed by the maternal LP diet, while a somewhat smaller number (967) of non-redundant transcripts, all encoding known genes, were changed in skeletal muscle (Table 2). Of the 2012 genes affected in the liver, 667 transcripts were up- and 1345 down-regulated. Similarly, in skeletal muscle; of the 967 genes affected, 312 were up- and 655 transcripts were down-regulated compared with a maternal NP diet (Table 2).

A number of significantly changed transcripts and one not significantly changed (CS) were randomly selected for RT-PCR validation of the microarray data. Positive validation was obtained for 7 out of 8 and 6 out of 9 transcripts encoding known genes in liver and skeletal muscle, respectively (see Additional file 1).

Taurine supplementation prevented a major part of the gene expression changes seen with the maternal LP diet (Table 2). Taurine had a rescuing effect on 30% (600 out of 2012) of the changed transcripts of known genes in liver and on 46% (444 out of 967) in skeletal muscle (Table 2), rendering the taurine effect more pronounced on muscle than on liver (p<10^-16^, Fishers exact test).

Among the changed genes, we found that mitochondrial genes and among those in particular genes involved in oxidative phosphorylation were over-represented in both liver and skeletal muscle (Table 3 and 4). Genes involved in the TCA cycle, pyruvate dehydrogenase and glycolysis were over-represented only in skeletal muscle (Table 4). Over-representation of genes involved in amino acid metabolism was observed both in liver and skeletal muscle (Table 3 and 4). However, the key regulator of mitochondrial biogenesis, peroxisome proliferator-activated receptor γ coactivator-1α  (PGC-1α), was downregulated by the LP diet in both tissues (see Additional file 1) and was not affected by taurine.

**Table 3:** Analysis of gene set over-representation in liver newborn mice.

		Ease		Taurine	Average fold change			
					
Category	Count	score	% Rescued	score	NP	NP+tau	LP	LP+tau
**All genes**	2012	na	30%	na	1	0.96	0.95	0.96
								
**KEGG pathway analysis**	620	na	31%	na	1	0.97	1.02	1.01
Oxidative phosphorylation	42	1.56E-06	43%	0.125	1	1.06	1.44	1.21
Valine, leucine and isoleucine degradation	17	3.13E-03	24%	0.604	1	1.04	1.47	1.27
Lysine degradation	17	4.12E-03	41%	0.428	1	1.02	1.40	1.24
Butanoate metabolism	15	2.04E-02	27%	1	1	0.99	1.16	1.19
Limonene and pinene degradation	10	2.06E-02	20%	0.732	1	0.98	1.15	1.14
								
**Panther biological pathways**	1965	na	30%	na	1	0.96	0.95	0.96
Fatty acid metabolism	122	7.90E-05	40%	0.027	1	0.95	0.86	0.94
Protein disulfide-isomerase reaction	90	1.16E-04	29%	0.907	1	0.99	1.04	1.01
Oxidative phosphorylation	175	1.63E-03	28%	0.606	1	0.94	0.97	0.97
Immunity and defense	171	2.34E-03	30%	1	1	0.99	0.86	0.95
Carbohydrate metabolism	86	4.54E-03	32%	0.722	1	0.97	1.07	1.05
								
**GO cellular component**	1627	na	30%	na	1	0.96	0.95	0.96
mitochondrion	209	4.71E-11	37%	0.057	1	1.03	1.29	1.13
Cytoplasm	866	4.53E-08	nd	nd	nd	nd	nd	nd
Intracellular part	1231	2.51E-07	nd	nd	nd	nd	nd	nd
mitochondrial inner membrane	68	1.21E-06	nd	nd	nd	nd	nd	nd
organelle inner membrane	69	3.63E-06	nd	nd	nd	nd	nd	nd

Genes found to have their expression level significantly changed in liver in newborn mice subjected to a maternal low protein diet compared to a normal diet were examined for over-representation of specific gene sets as described. The top 5 most significant over-represented gene sets are shown. Count) Total number of genes in analysis. Ease score) a modified fishers exact test describing the probability of gene set enrichment. % Rescued) Amount of rescued or partially rescued genes (see table 2 and materials and methods) in percent of count. Taurine score) Fishers exact test examining if taurine preferentially rescued the gene set compared to the rescue effect seen in all genes examined in the parent geneset. Average fold change) Means of fold change for the 4 diet groups per gene set in regard to NP. NP) normal protein (20% casein) or LP) low protein (8% casein) during pregnancy, tau) 1% taurine supplementation in the drinking water during pregnancy. na) not applicable. nd) not determined. Table reprinted with permission from Wolters Kluwer Health / Lippincott Williams & Wilkins. [52]

**Table 4:** Analysis of gene set over-representation in skeletal muscle in newborn mice.

		Ease		Taurine	Average fold change			
					
Category	Count	score	% Rescued	score	NP	NP+tau	LP	LP+tau
**All genes**	967	na	46%	na	1	0.98	0.95	0.94
								
**KEGG pathway analysis**	263	na	46%	na	1	0.99	0.90	0.93
Oxidative phosphorylation	24	5.60E-06	75%	0.010	1	0.92	0.72	1.04
Citrate cycle (TCA cycle)	8	2.45E-03	88%	0.029	1	0.96	0.58	0.93
Valine, leucine and isoleucine degradation	10	4.53E-03	40%	0.757	1	1.02	0.59	0.81
Pyruvate metabolism	9	9.62E-03	67%	0.314	1	0.99	0.49	0.86
Glycolysis / Gluconeogenesis	10	1.12E-02	60%	0.523	1	0.96	0.61	0.98
								
**Panther biological pathways**	928	na	46%	na	1	0.98	0.94	0.94
Electron transport	65	3.99E-04	45%	1	1	0.96	0.84	0.98
Muscle contraction	13	5.12E-03	64%	0.189	1	0.84	0.38	0.84
Cell adhesion-mediated signaling	33	5.85E-03	52%	0.596	1	0.99	1.06	0.99
Cell motility	48	9.44E-03	44%	0.769	1	1.00	1.16	0.97
Ion transport	133	1.21E-02	53%	0.164	1	0.97	0.94	0.93
								
**GO cellular component**	777	na	47%	na	1	0.98	0.94	0.94
Mitochondrion	121	2.46E-13	56%	0.065	1	0.97	0.62	0.91
cytoplasmic part	288	8.48E-10	nd	nd	nd	nd	nd	nd
Cytoplasm	441	1.57E-09	nd	nd	nd	nd	nd	nd
mitochondrial part	56	4.70E-09	nd	nd	nd	nd	nd	nd
mitochondrial inner membrane	42	1.12E-07	nd	nd	nd	nd	nd	nd

Genes found to have their expression level significantly changed in skeletal muscle in newborn mice subjected to a maternal low protein diet compared to a normal diet were examined for over-representation of specific gene sets as described. The top 5 most significant over-represented gene sets are shown. Count) Total number of genes in analysis. Ease score) a modified fishers exact test describing the probability of gene set enrichment. % Rescued) Amount of rescued or partially rescued genes (see table 2 and materials and methods) in percent of count. Taurine score) Fishers exact test examining if taurine preferentially rescued the gene set compared to the rescue effect seen in all genes examined in the parent geneset. Average fold change) Means of fold change for the 4 diet groups per gene set in regard to NP. NP) normal protein (20% casein) or LP) low protein (8% casein) during pregnancy, tau) 1% taurine supplementation in the drinking water during pregnancy. na) not applicable. nd) not determined. Table reprinted with permission from Wolters Kluwer Health / Lippincott Williams & Wilkins. [52]

**Figure 1:**
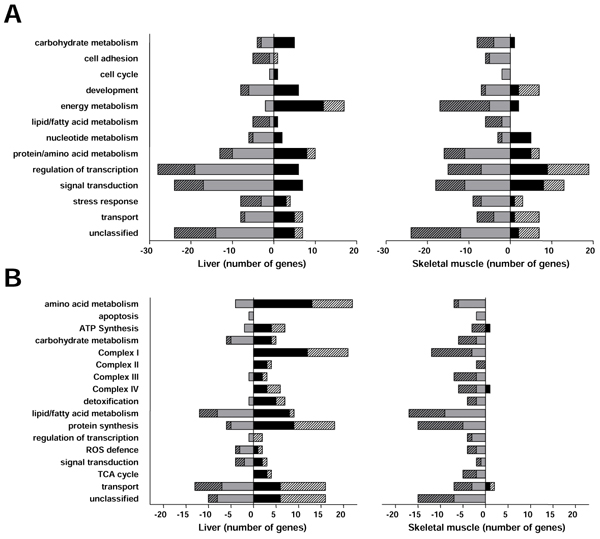
**Classification of changed genes by biological function** A) Genes significantly changed in newborn mice in response to an *in utero* low protein diet in liver and skeletal muscle. For comparison, only genes that were changed in both liver and skeletal muscle are shown. B) Mitochondrial genes changed in newborn mice in response to an *in utero* low protein diet in liver and skeletal muscle. Grey/black bars indicate down/up-regulated genes in newborn mice subjected to a low protein diet in utero compared to a normal protein diet. Hatched bars indicate the number of genes rescued by taurine supplementation. Figure reprinted with permission from Wolters Kluwer Health / Lippincott Williams & Wilkins. [52]

When genes were grouped according to function (figure 1A) (see Additional file 2), a large difference was seen between up and down-regulated genes involved in energy metabolism (figure 1A) in both liver and skeletal muscle., albeit in a different direction in the two tissues. Likewise, when mitochondrial genes were grouped according to function, a clear difference between liver and skeletal muscle could be seen (figure 1B) (see Additional file 3 and 4). The rescuing effect of taurine was significantly higher on genes involved in fatty acid metabolism in liver compared to other gene sets (Table 3), whereas in skeletal muscle this rescuing effect was present only for genes involved in oxidative phosphorylation and the TCA cycle (Table 4).

### Gestational taurine supplementation has a lasting effect

Since taurine primarily affected mitochondrial genes changed by the gestational low protein diet, it is of interest to note that we did not find any effect of taurine on the newborn offspring of dams fed an NP diet during gestation. However, in 4-week-old offspring a subset of mitochondrial genes involved in the mitochondrial ribosome function were upregulated in liver (Table 5). In muscle there was an increase (SAM, FDR < 10%) in 11 mitochondrial genes, including genes involved in lipid metabolism (carnitine palmitoyl transferase 2), methionine metabolism (Methionine sulfoxide reductase B2) and mitochondrial rRNA methylation (mitochondrial rRNA methyltransferase 1), (Table 6).

**Table 5:** The effect of gestational taurine supplementation on gene set enrichment analysis of 4 week old mice

Category / tissue	Size	ES	NES	FDR	FWER
**GO cellular component**					
*Liver*					
Mitochondrial ribosome	21	0.58	1.90	0.012	0.038
Microbody	43	0.48	1.92	0.014	0.035
Peroxisome	43	0.48	1.96	0.016	0.026
Organellar ribosome	21	0.58	1.99	0.023	0.018
Ribosomal subunit	19	0.53	1.79	0.032	0.116
*Muscle*					
Pore complex	33	0.51	1.50	0.017	0.917
Proteasome complex	22	0.53	1.48	0.020	0.948
Apicolateral plasma membrane	15	-0.60	-1.78	0.043	0.102
Voltage gated potassium channel complex	22	-0.56	-1.89	0.052	0.043
Extracellular region	236	-0.32	-1.68	0.060	0.218
					
**KEGG**					
*Liver*					
Biosynthesis of steroids	22	0.81	2.79	0.000	0.000
Hematopoietic cell lineage	45	-0.62	-1.86	0.007	0.006
Fatty acid metabolism	37	0.48	1.81	0.026	0.057
Butanoate metabolism	37	0.45	1.76	0.029	0.083
Glycerolipid metabolism	39	0.46	1.81	0.038	0.056
*Muscle*					
Metabolism of xenobiotics by cytochrome P450	23	-0.64	-2.24	0.001	0.001
Complement and coagulation cascades	34	-0.52	-1.91	0.007	0.024
Ribosome	54	-0.46	-1.93	0.009	0.021
Neuroactive ligand receptor interaction	86	-0.41	-1.83	0.010	0.046
Alzheimers disease	23	-0.51	-1.76	0.019	0.100

Gene set enrichment analysis (GSEA) was performed in order to detect differences between liver  and skeletal muscle of 4 week old mice subjected to taurine supplementation during gestation as described in materials and methods. The top 5 most significant genesets (based on FDR) is shown. Size) total number of genes in analysis from the geneset. ES) Enrichment score, reflects the degree to which a gene set is overrepresented. NES) Normalized enrichment score, is an ES score that is comparable between genesets. A positive value for ES and NES indicates an upregulation of said geneset by taurine, likewise a negative value indicates a downregulation of the geneset in question. FDR) False discovery rate. FWER) Family-wise error rate.

**Table 6:** Mitochondrial genes significantly changed by gestational taurine supplementation in skeletal muscle of 4 week old mice

Probe	Gene symbol	Gene name	Fold change
1428487_s_at	COQ10B	coenzyme Q10 homolog B	3.04
1416772_at	Cpt2	Carnithine palmitoyltransferase 2	1.56
1425140_at	Lactb2	Lactamase, beta 2	1.81
1424219_at	MRM1	Mitochondrial rRNA methyltransferase 1	1.78
1451266_at	Mrpl50	Mitochondrial ribosomal protein L50	1.65
1424433_at	Msrb2	Methionine sulfoxide reductase B2	2.21
1449897_a_at	MTCP1	Mature T-cell proliferation 1	1.55
1445632_at	OGDH	Oxoglutarate dehydrogenase (lipoamide)	1.52
1452676_a_at	PNPT1	Polyribonucleotide nucleotidyltransferase 1	1.56
1424735_at	Slc25a25	Solute carrier family 25 (phosphate carrier), member 25	2.48
1425753_a_at	Ung	Uracil DNA glycosylase	1.93

A list of probes and corresponding gene symbols and names for mitochondrial genes significantly changed by gestational taurine supplementation. Fold change indicates the fold change increase in the taurine supplemented group (NP+tau). vs. the non-treated group (NP).

## Discusson

The novel findings of this study were 1) that a maternal low protein diet induced substantial changes in gene expression patterns in newborn offspring in both liver and skeletal muscle, 2) that the changes in mitochondrial genes in general and genes of oxidative phosphorylation in particular were prominent in both liver and skeletal muscle, with predominantly upregulation in liver and downregulation in skeletal muscle, 3) that maternal taurine supplementation in the drinking water during pregnancy to a surprisingly large extent prevented these changes in gene expression patterns in both liver and skeletal muscle, and finally 4) that gestational taurine supplemention of control dams during gestation seemed to have an effect upon mitochondrial gene expression in the offspring at 4 weeks of age.

### Birthweight

The 40% decrease in birthweight of LP offspring observed in this study  is somewhat larger than previously reported in the same mouse strain (7% and 28%) [32]. We observed no difference in the ratio of organ weight:total body weight, which seems to disagree with a previous study  in rats [33], and we saw a large rescue effect of taurine upon birthweight, in disagreement with a similar study in rats [19]. However, these disagreements likely reflect species or diet composition differences.

### Changes in global gene expression patterns in liver and skeletal muscle

Genes involved in oxidative phosphorylation and other mitochondrial genes were the most over-represented in the genes changed in the LP offspring (Table 3 and 4). This suggests that dysregulation of energy metabolism is a key component in the development of the low birthweight phenotype. Furthermore, as CS activity may be considered a measure of mitochondrial mass, the lack of a change in CS activity suggests this dysregulation may happen without a change in mitochondrial mass. However, this seem to be at variance with Park et al. [34], who reported a decreased mtDNA:gDNA ratio in both liver and skeletal muscle in 5-week-old offspring of maternal low protein rats.

In liver, a large fraction of genes involved in amino acid metabolism, protein synthesis, TCA cycle and energy metabolism genes involved in both Complex I-IV and ATP synthesis showed an increased expression, whereas in skeletal muscle the opposite effect was observed. The concentration of taurine in skeletal muscle is ~6 fold higher than in the liver in mice [35], suggesting a different taurine requirement of the two tissues. It should also be noted that a decrease in taurine availability is known to occur under LP conditions [36], and that taurine is a constituent of mitochondrial tRNA [13] and may thus be required for normal mitochondrial function.

Downregulation of oxidative phosphorylation in skeletal muscle has in several cases been linked to development of type 2 diabetes and insulin resistance [37,38]. Although the exact mechanism is unknown, a decrease in the number of skeletal muscle mitochondria and/or in the activity of oxidative phosphorylation as well as mitochondrial dysfunction at birth may, if it is permanent, be associated with a decreased capacity for β-oxidation capacity, which may lead to increased intracellular lipid content and subsequent disruption of insulin signaling [39]. However, this hypothesis has recently been questioned [40].

Interestingly, in humans birthweight is positively associated with the expression of PGC-1α in skeletal muscle in adults [6], but despite PGC-1α controlling mitochondrial biogenesis [41], no concomitant difference in mitochondrial gene expression and oxidative phosphorylation was observed in adults with a low birthweight [6]. However, the decrease in mitochondrial gene expression seen in this study and not in adult humans may reflect the difference between newborns and adults or species difference.

### Partial phenotype rescue by taurine supplementation

Taurine has previously been shown to rescue the adverse effects of a maternal LP diet on pancreatic function in the offspring [19,20,23,42]. We find a large rescuing effect on gene expression profiles in both liver and muscle by taurine supplementation, with significantly more mitochondrial genes rescued in skeletal muscle than in liver, suggesting taurine as an important factor in the control of mitochondrial gene expression. Furthermore, we observed that the rescuing effect was tissue specific, as genes of fatty acid metabolism in the liver and genes involved in oxidative phosphorylation and TCA cycle in skeletal muscle were preferentially rescued compared with other genes (Table 3 and 4).  A similar study examining the effect of maternal LP diet and taurine on islet gene expression also found decreased expression of respiration and TCA cycle genes, changes which were rescued fully by taurine supplementation [23]. Combined with our results, this strongly suggests that the effect exerted by taurine is mediated by a mitochondrial mechanism.

In this context it should be noted that plasma taurine concentration has been suggested to be a marker of fetal well being and a prerequisite for normal fetal development [43]. This is corroborated by the observation that lack of the taurine transporter (TAUT) confers a large decrease in exercise capacity [14], which may also be related to the observation that TAUT expression increases during myogenesis and that taurine is able to protect against dexamethasone induced atrophy [44]. Thus, a picture of taurine as a necessary expression factor in myogenesis, and perhaps in mitochondrial biogenesis as well, emerges.

In liver a deficiency of taurine, as seen in the TAUT knockout mouse, causes an increase in hepatic apoptosis and inflammation, as well as a decrease in mitochondrial coupling of oxidative phosphorylation [45]. Also, taurine has been shown to have an anti-apoptotic effect upon the liver and to normalize tamoxifen induced mitochondrial dysfunction in rats [46]. There are several reports on the effect of a maternal low protein diet on offspring DNA methylation patterns [47,48], but there is no evidence of taurine involvement in this process. Interestingly, taurine has been shown to decrease N-methylation in rat heart [49].

Taurine supplementation during gestation alone, with no other dietary manipulations of the maternal diet, resulted in minute changes to mitochondrial gene expression in both skeletal muscle and liver, primarily in lipid metabolism and protein translation. Others have shown both beneficial [50] and detrimental effects [51] of gestational taurine supplementation upon the adult metabolic phenotype, however no mechanism for these changes have been identified. The observation that gestational taurine supplementation also in the control group has an effect on mitochondrial gene expression and lipid metabolism corroborates the notion of taurine as being important for development of normal mitochondrial function, a field of research which needs more attention.

## Conclusion

A low protein diet to mice during pregnancy caused major changes in both body mass and gene expression profiles of liver and skeletal muscle of the newborn mice. The expression changes were predominantly related to mitochondrial genes but were markedly different in liver and skeletal muscle. Maternal taurine supplementation in the drinking water partially prevented both the change in body mass and changes in gene expression. In particular, skeletal muscle genes involved in oxidative phosphorylation were almost completely normalized by the taurine supplementation. The mechanism of these taurine effects remains unknown, but it may be suggested that taurine is a factor controlling mitochondrial gene expression during development, possibly by an epigenetic mechanism.

## Competing interests

The authors declare that they have no competing interests.

## List of abbreviations

CS: citrate synthase; LP: low protein ; NP: normal protein; PGC-1α: peroxisome proliferator-activated receptor γ coactivator-1α; Tau: taurine; TCA: tricarboxylic acid.

## Authors’ contributions

HLO and LF  performed animal experimental work, RNA isolation, preliminary microarray analyses and enzyme assays. FCN performed the bulk of the microarray experiments. OHM carried out the bioinformatic and statistical analyses and validated genes. PEN, NG and BQ conceived and designed the study and contributed to data analysis. OHM wrote the first draft of the manuscript which was finalized i collaboration with NG and BQ. All authors read and approved the final manuscript.

## Supplementary Material

Additional file 1 Mortensen OH LP tau mice additional file 1.pdf, Adobe pdf document. Supplemental table 1. Validation of genes significantly changed in newborn mice subjected to a maternal low protein diet. Click here for file

Additional file 2 Mortensen OH LP tau mice additional file 2.xls, Excel spreadsheet. Supplemental table 2. Genes significantly different in both liver and skeletal muscle of newborn mice subjected to either a maternal low protein diet or a maternal normal protein diet as described. L: Liver, M: Skeletal muscle. Fully rescued genes (FR), partially rescued genes (PR). All expression values are represented as a single value of fold-change compared to NP. Click here for file

Additional file 3 Mortensen OH LP tau mice additional file 3.xls, Excel spreadsheet. Supplemental table 3. Significantly changed mitochondrial genes in liver of newborn mice. All expression values are represented as a single value of fold-change compared to NP. Click here for file

Additional file 4 Mortensen OH LP tau mice additional file 4.xls, Excel spreadsheet. Supplemental table 4. Significantly changed mitochondrial genes in skeletal muscle of newborn mice. All expression values are represented as a single value of fold-change compared to NP. Click here for file
