# Supplementary material for: A maternal low protein diet has pronounced effects on mitochondrial gene expression in offspring liver and skeletal muscle; protective effect of taurine
Source: J Biomed Sci. 2010 Aug 24;17(Suppl 1):S38. doi: 10.1186/1423-0127-17-S1-S38 (PMC2994375; doi:10.1186/1423-0127-17-S1-S38)
Supplement: Additional file 1 — Mortensen OH LP tau mice additional file 1.pdf, Adobe pdf document. Supplemental table 1. Validation of genes significantly changed in newborn mice subjected to a maternal low protein diet. [file 1423-0127-17-S1-S38-S1.pdf]

Supplemental table 1A - liver

Taurine	-	+	-	+	2-way ANOVA		
Protein	NP	NP	LP	LP	chow	taurine	c*t
ACSS2	1.00 (0.79-1.27)	1.02 (0.79-1.33)	0.51 (0.37-0.70)[¶]	0.83 (0.72-0.96)[‡]	0.0001	0.0108	0.0183
1422478_a_at	1.00 (0.66-1.51)	0.56 (0.30-1.03)	0.10 (0.08-0.14)	0.45 (0.27-0.78)	0.0010	ns	0.0030
CS	1.00 (0.90-1.11)	1.05 (0.95-1.16)	0.91 (0.80-1.04)	1.02 (0.92-1.12)	ns	ns	ns
1450667_a_at	1.00 (0.89-1.12)	0.92 (0.64-1.32)	0.65 (0.55-0.78)	0.95 (0.82-1.11)	ns	ns	ns
EGFR	1.00 (0.90-1.12)	1.06 (0.94-1.19)	0.79 (0.61-1.02)	0.87 (0.72-1.05)	0.0079	ns	ns
1435888_at	1.00 (0.67-1.50)	0.61 (0.30-1.28)	0.07 (0.02-0.24)	0.54 (0.24-1.24)	0.0120	ns	0.0186
FST	1.00 (0.82-1.23)	1.32 (1.04-1.66)	0.43 (0.32-0.57)[¶]	0.89 (0.69-1.15)[‡, §]	0.0001	0.0001	0.0493
1434458_at	1.00 (0.69-1.45)	0.73 (0.57-0.94)	0.24 (0.16-0.36)	1.20 (0.76-1.89)	0.0424	0.0102	0.0011
GLDC	1.00 (0.89-1.13)	1.02 (0.82-1.28)	1.60 (0.84-3.01)	0.95 (0.67-1.34)	ns	ns	(0.0797)
1416049_at	1.00 (0.72-1.39)	1.07 (0.79-1.47)	3.22 (2.97-3.48)	2.03 (1.37-3.03)	0.0004	ns	ns
GPD2	1.00 (0.88-1.14)	0.99 (0.88-1.13)	0.75 (0.57-0.97)[*]	1.00 (0.87-1.15)[†]	0.0417	0.0415	0.0331
1417434_at	1.00 (0.75-1.34)	1.02 (0.76-1.36)	0.43 (0.35-0.53)	1.05 (0.81-1.36)	0.0174	0.0100	0.0120
PGC-1α	1.00 (0.83-1.18)	1.23 (0.94-1.75)	0.79 (0.56-1.12)	0.86 (0.40-1.57)	0.0096	ns	ns
1434100_x_at	1.00 (0.86-1.14)	0.50 (0.29-0.86)	0.48 (0.32-0.73)	0.80 (0.60-1.06)	ns	ns	0.0141
SPP1	1.00 (0.81-1.24)	0.84 (0.69-1.02)	0.53 (0.34-0.83)	0.70 (0.43-1.15)	ns	ns	ns
1449254_at	1.00 (0.87-1.15)	1.34 (0.96-1.87)	0.19 (0.13-0.30)	1.19 (0.55-2.57)	0.0068	0.0025	0.0141

Supplemental table 1B – skeletal muscle

Taurine	-	+	-	+	2-way ANOVA		
Protein	NP	NP	LP	LP	chow	taurine	c*t
COX7A1	1.00 (0.80-1.25)	0.93 (0.73-1.19)	0.54 (0.39-0.74)[**]	0.76 (0.61-0.96)	0.0005	ns	0.0475
1418709_at	1.00 (0.78-1.28)	0.89 (0.64-1.23)	0.21 (0.14-0.30)	1.15 (0.94-1.41)	0.0024	0.0008	0.0003
COX7C	1.00 (0.72-1.39)	0.83 (0.59-1.17)	0.76 (0.44-1.29)	0.78 (0.64-0.95)	ns	ns	ns
1459884_at	1.00 (0.93-1.09)	0.86 (0.80-0.92)	2.43 (1.56-3.76)	1.39 (0.79-2.44)	0.0058	(0.0896)	ns
CS	1.00 (0.76-1.32)	1.02 (0.72-1.43)	0.83 (0.67-1.03)	0.82 (0.65-1.02)	(0.0728)	ns	ns
1450667_a_at	1.00 (0.90-1.11)	0.92 (0.67-1.26)	0.65 (0.56-0.76)	0.95 (0.83-1.09)	(0.0871)	ns	0.0499
GPD2	1.00 (0.69-1.46)	1.04 (0.72-1.48)	0.80 (0.66-0.96)	0.80 (0.63-1.03)	(0.0651)	ns	ns
1417434_at	1.00 (0.85-1.17)	0.96 (0.79-1.18)	0.62 (0.58-0.67)	1.26 (1.04-1.53)	ns	0.0040	0.0022
MH1	1.00 (0.74-1.35)	0.94 (0.74-1.21)	0.63 (0.41-0.96)	0.69 (0.44-1.08)	0.0135	ns	ns
1427868_x_at	1.00 (0.77-1.30)	0.85 (0.47-1.51)	0.33 (0.28-0.38)	1.00 (0.68-1.47)	0.0396	0.0385	0.0102
MYBPC2	1.00 (0.75-1.33)	0.91 (0.68-1.20)	0.21 (0.14-0.34)[¶]	0.50 (0.27-0.91)[*,†]	0.0001	0.0452	0.0139
1455736_at	1.00 (0.96-1.04)	0.76 (0.54-1.06)	0.04 (0.03-0.07)	0.56 (0.27-1.15)	0.0001	0.0010	0.0002
PDK4	1.00 (0.74-1.36)	0.87 (0.59-1.29)	0.38 (0.30-0.48)	0.41 (0.26-0.65)	0.0001	ns	ns
1417273_at	1.00 (0.70-1.43)	0.70 (0.26-0.92)	0.12 (0.09-0.17)	0.33 (0.15-0.76)	0.0039	ns	(0.0931)
PGC-1α	1.00 (0.70-1.75)	1.10 (0.56-2.19)	0.62 (0.44-0.90)	0.68 (0.32-1.13)	0.0084	ns	ns
1456395_at	1.00 (0.59-1.70)	1.27 (0.46-3.45)	0.32 (0.22-0.47)	1.31 (0.72-2.38)	ns	0.0426	ns
UCP3	1.00 (0.73-1.37)	0.75 (0.47-1.20)	0.34 (0.19-0.60)	0.40 (0.28-0.56)	0.0001	ns	ns
1420658_at	1.00 (0.68-1.47)	0.89 (0.51-1.53)	0.31 (0.28-0.34)	0.54 (0.31-0.97)	0.0062	ns	ns

Supplemental table 1. *Validation of genes significantly changed in newborn mice subjected to a maternal low protein diet*

Quantitative RT-PCR determined mRNA levels of selected significantly changed genes and citrate synthase together with the respective expression levels from the microarray probes. All values are shown as geometric means with 95% confidence intervals normalized to control (NP), with $5 \leq N \leq 7$ per group (quantitative RT-PCR mRNA levels) and N=3 per group (microarray probes). The results of a two-way ANOVA testing for effect of chow and taurine as well as

the interaction are shown. ACSS2 (acyl-CoA synthetase short-chain family member 2), COX7A1 (cytochrome c oxidase, subunit VIIa 1), COX7C (cytochrome c oxidase, subunit VIIc), CS (citrate synthase), EGFR (epidermal growth factor receptor), FST (Follistatin), GLDC (glycine decarboxylase), GPD2 (glycerol phosphate dehydrogenase 2, mitochondrial), MH1 (myosin, heavy polypeptide 1, skeletal muscle, adult), MYBPC2 (myosin binding protein C, fast-type), PDK4 (pyruvate dehydrogenase kinase, isoenzyme 4), SPP1 (secreted phosphoprotein 1), UCP3 (uncoupling protein 3). A) liver, B) skeletal muscle. *) $p<0.05$, **) $p<0.01$, ¶) $p<0.001$ significantly different from NP without taurine. †) $p<0.05$, ‡) $p<0.01$ significantly different from LP without taurine. §) significantly ($p<0.05$) different from NP with taurine. Table reprinted with permission from Wolters Kluwer Health / Lippincott Williams & Wilkins (Mortensen OH et al. Pediatr Res 2010, 67: 47-53)
